# Neuroimaging Insight Into Fragile X-Associated Neuropsychiatric Disorders: Literature Review

**DOI:** 10.3389/fpsyt.2021.728952

**Published:** 2021-10-15

**Authors:** Andrea Elias-Mas, Maria Isabel Alvarez-Mora, Conxita Caro-Benito, Laia Rodriguez-Revenga

**Affiliations:** ^1^Radiology Department, Hospital Universitari Mútua de Terrassa, Terrassa, Spain; ^2^Institute for Research and Innovation Parc Taulí (I3PT), Sabadell, Spain; ^3^Universitat Internacional de Catalunya (UIC), Barcelona, Spain; ^4^Biochemistry and Molecular Genetics Department, Hospital Clinic of Barcelona, Barcelona, Spain; ^5^CIBER of Rare Diseases (CIBERER), Instituto de Salud Carlos III, Barcelona, Spain; ^6^Institut d'Investigacions Biomèdiques August Pi i Sunyer (IDIBAPS), Barcelona, Spain; ^7^Hospital Library, Research Foundation Mutua Terrassa, Barcelona, Spain

**Keywords:** FXAND, neuropathology, *FMR1* premutation, neuroimaging, functional studies

## Abstract

*FMR1* premutation is defined by 55–200 CGG repeats in the Fragile X Mental Retardation 1 (*FMR1*) gene. *FMR1* premutation carriers are at risk of developing a neurodegenerative disease called fragile X-associated tremor/ataxia syndrome (FXTAS) and Fragile X-associated primary ovarian insufficiency (FXPOI) in adulthood. In the last years an increasingly board spectrum of clinical manifestations including psychiatric disorders have been described as occurring at a greater frequency among *FMR1* premutation carriers. Herein, we reviewed the neuroimaging findings reported in relation with psychiatric symptomatology in adult *FMR1* premutation carriers. A structured electronic literature search was conducted on *FMR1* premutation and neuroimaging yielding a total of 3,229 articles examined. Of these, 7 articles were analyzed and are included in this review. The results showed that the main radiological findings among adult *FMR1* premutation carriers presenting neuropsychiatric disorders were found on the amygdala and hippocampus, being the functional abnormalities more consistent and the volumetric changes more inconsistent among studies. From a molecular perspective, CGG repeat size, *FMR1* mRNA and FMRP levels have been investigated in relation with the neuroimaging findings. Based on the published results, FMRP might play a key role in the pathophysiology of the psychiatric symptoms described among *FMR1* premutation carriers. However, additional studies including further probes of brain function and a broader scope of psychiatric symptom measurement are required in order to obtain a comprehensive landscape of the neuropsychiatric phenotype associated with the *FMR1* premutation.

## Introduction

Fragile X premutation carriers is defined by 55–200 CGG repeats in the Fragile X Mental Retardation 1 (*FMR1*) gene, whilst the full mutation is caused by >200 CGG repeats. Two major conditions associated with the *FMR1* premutation have been well-established: the fragile X-associated Tremor/Ataxia Syndrome (FXTAS) and the fragile X-associated Primary Ovarian Insufficiency (FXPOI) [reviewed in (1)] FXTAS is a late onset neurodegenerative condition, and it is seen in around 40% of male premutation carriers and 16% of females (2). FXPOI is characterized by menopause before age 40 and it is seen in around 20% of women with the *FMR1* premutation (3). Nevertheless, a broader clinical spectrum of symptoms, including psychiatric, sleep, and autoimmune conditions, has been described among *FMR1* permutation carriers (4). Although the extent of all this group of conditions needs further delineation, in order to bring recognition to these problems, different names were proposed. Hagerman et al., proposed fragile X-associated Neuropsychiatric Disorders (FXAND) and the European Fragile X Network (EFXN) proposed to name them Fragile X-associated Neuropsychiatric Conditions (FXANC), and Fragile X Various Associated Conditions (FXVAC) to cover other physical conditions associated with the *FMR1* premutation (51).

*FMR1* premutation main neuropsychiatric disorders in adults include anxiety and depression and, to a lesser extent, obsessive compulsive disorder (OCD), attention-deficit/hyperactivity disorder (ADHD), and substance abuse. Although most prior work has been performed examining different neuropsychiatric aspects in separate study samples, neuroimaging studies have depicted structural, functional, and connectivity changes within the brain associated to neuropsychiatric disorders. In regard to *FMR1* premutation this relationship has been addressed, although with different approaches and with different population cohorts. Furthermore, a correlation on how neurostructural and neurofunctional effects might be associated with molecular aspects of the *FMR1* premutation and psychiatric symptomatology has also been reported with varying results. This review focuses on the neuroimaging findings associated with neuropsychiatric disorders in adult *FMR1* premutation carriers. Due to the wide clinical spectrum of neurological symptoms associated with the *FMR1* premutation, including motor and cognitive impairment, executive and memory deficits, and psychiatric symptoms, we believe that a review focusing on the neuropathology, molecular underpinnings, and neuroimaging associated with neuropsychiatric findings could help to untangle the complex physiopathology associated with the *FMR1* premutation.

## Methods

### Search Strategy

A review of the literature was conducted. PubMed, Web of Science, Pschynfo and Cochrane Central Register of Controlled Trials were searched for eligible studies from 2000 to April 2021. Search terms are listed in Research Algorithm (Supplementary Table 1) where the complete search strategy is displayed. Inclusion criteria included *FMR1* premutation carriers, both genders, from 18 to 99 years old and all ethnicities. As exclusion criteria, the search was only focused on pure psychiatric symptoms and therefore motor and cognitive impairments, executive function, or memory deficits were not considered.

### Study Eligibility Criteria

**Figure 1:**
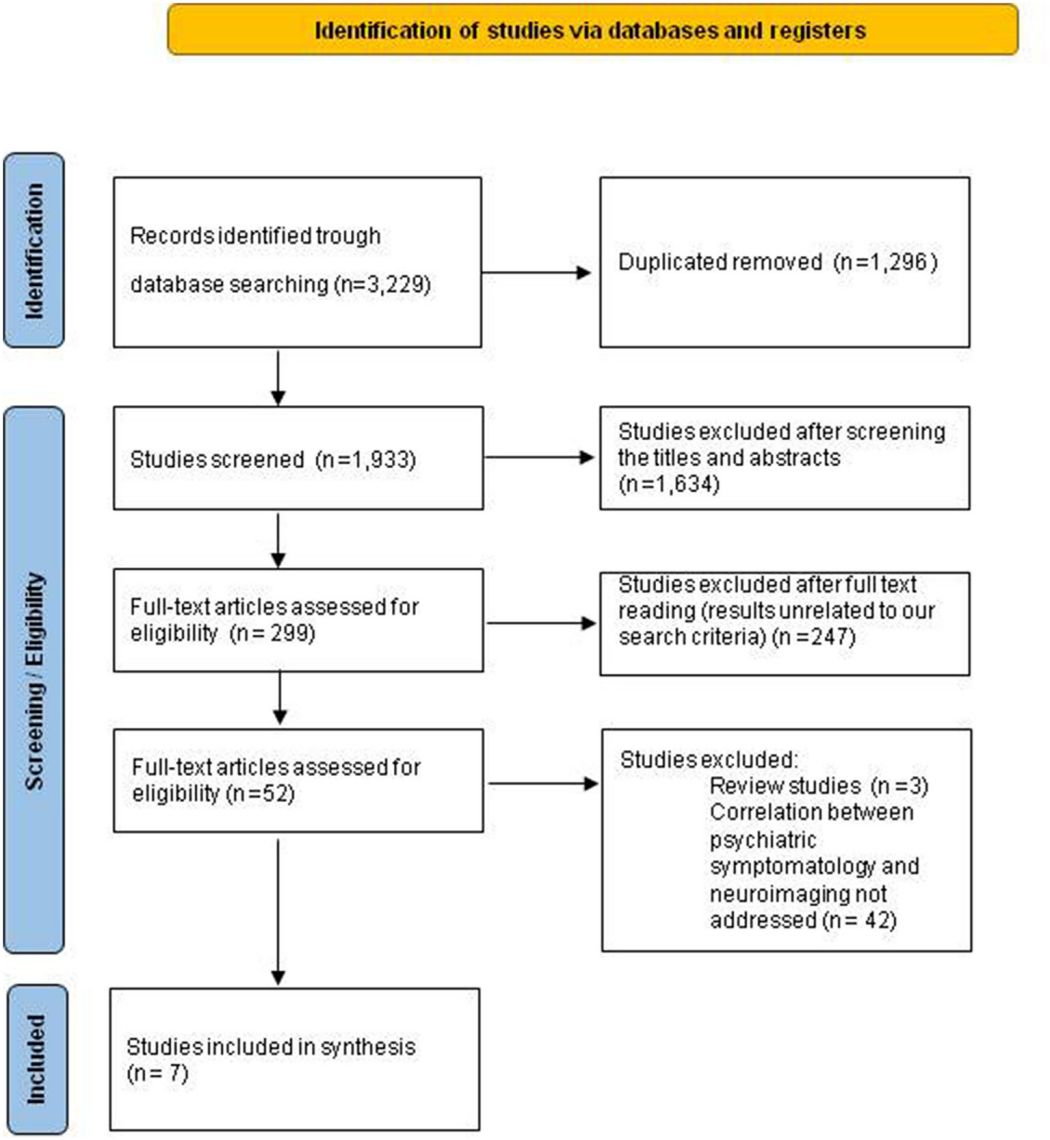
Diagram for identification and selection of studies.

Eligible qualitative studies were those that matched neuroimaging findings with psychiatric symptomatology in *FMR1* premutation carriers. The type of studies selected were those of any design published in peer-reviewed academic journals with abstract available. Conference proceedings, theses, case reports and case series were excluded, and review articles, non-English articles, and studies on animal models were not considered (for a graphical summary of the selection procedure, see Figure 1).

After establishing and applying the inclusion criteria, two researchers (A. EM and L.RR) separately read the titles of the papers retrieved in the search and examined the structured abstract of the selected articles. Seven studies that described the relationship between neuroimaging and neuropsychiatric findings in adult *FMR1* premutation were selected. All potential differences in interpretation between the reviewers were discussed to ensure that all the articles reviewed presented a satisfactory level of evidence.

This study is a review of previously published data and, as such, does not require ethics approval. The data were not used for any purpose other than those of the original study, and no new data were collected.

## Results

### Synthesis of Qualitative Studies

A total of 3,229 studies were identified from the initial search. After de-duplication and initial screening, 1,933 full-text articles were assessed for eligibility and 7 were included in this review (Figure 1, Table 1) (5–11).

**Table 1:** Summary of the reviewed studies.

**Author and Year**	**Participants**	**Cognitive test**	**Psychological test**	**Molecular measures**	**Imaging (MRI)**	**Other**
Hessl et al. (5)	12 *FMR1* premutation males without FXTAS 13 male controls	WAIS-III	SCL-90-R	CGG repeat size *FMR1* mRNA	Amygdala volumes Total cranial volume Total brain volume fMRI face processing task	Eye blink response to fear-potentiated startle (EMG activity of orbicularis oculi) Skin conductance during social greeting (electrodermal electrodes)
Koldewyn et al. (6)	11 *FMR1* premutation males without FXTAS 11 male controls	WAIS-III	SCL-90-R	CGG repeat size *FMR1* mRNA	fMRI recall task Total brain volume Hippocampal volume	-
Adams et al. (7)	16 *FMR1* premutation female with FXTAS 17 *FMR1* premutation female without FXTAS 8 female controls 34 *FMR1* premutation male with FXTAS 21 *FMR1* premutation male without FXTAS 30 male controls	WAIS-III	SCL-90-R	CGG repeat size *FMR1* mRNA methylation status activation ratio	Total brain volume Hippocampal volume	FXTAS clinical staging
Hashimoto et al. (8)	31 *FMR1* premutation male with FXTAS 24 *FMR1* premutation male without FXTAS 28 male controls	Behavioral Dyscontrol Scale 2 Working Memory Score of the WAIS-III	SCL-90-R	CGG repeat size *FMR1* mRNA	Voxel-based morphometry analysis	FXTAS clinical staging
Hessl et al. (9)	23 *FMR1* premutation male without FXTAS 25 male controls	WAIS-III	Autism Diagnostic Observation Schedule Module 4 SRS (adult version)	CGG repeat size *FMR1* mRNA methylation status FMRP levels	Amygdala volume Total cerebral volume fMRI targeting the amygdala with an emotion processing task and concurrent infra-red eye tracking	-
Selmeczy et al. (10)	49 *FMR1* premutation male without FXTAS 48 male controls	WAIS-III	SCL-90-R	CGG repeat size *FMR1* mRNA	Amygdala volume Total cerebral volume	-
Brown et al. (11)	17 *FMR1* premutation male without FXTAS 17 male controls	KBIT	SCL-90-R AQ EQ Ekman Faces Test (version 1.0)	CGG repeat size	Emotional processing fMRI task to examine the response to a change in emotional arousal	-

*SCL-90-R, Symptom Checklist-90—Revised; WAIS-III, Wechsler Adult Intelligence Scale (Third Edition); KBIT, Kaufman Brief Intelligence Test (Second Edition); AQ, Autism Spectrum Quotient; EQ, Empathy Quotient; SRS, Social Responsiveness Scale*.

Represented in these manuscripts were views from 413 study participants (244 *FMR1* premutation carriers and 169 control individuals) from 2 countries (UK, USA). Participants included adult men and women ranging in age from 18 to over 79 years. *FMR1* premutation carriers were recruited through screening of pedigrees of probands with FXS with a CGG repeat size ranging from 55 CGGs to 199 CGGs. All subjects who participated in Koldewyn et al. (6) also participated in the Hessl et al. (5) study and, therefore, were counted only once. Such relation was not mentioned in the rest of the studies. One study (11) included 3 *FMR1* premutation mosaic participants and 1 intermediate allele participant within the premutation group and, thus, was not considered when evaluating the CGG range (Table 2).

**Table 2:** Study participant characteristics.

**Study (Country)**	**Control population**					***FMR1*** **premutation**				
	**Number of participants**	**Age (years old)**	**Ethnicity**	**CGGs repeat size**	**Medical history**	**Number of participants**	**Age (years old)**	**Ethnicity**	**CGGs repeat size**	**Medical history**
Hessl et al. (5) (USA)	*N* = 11 (all men)	26–55	2 Hispanic 1 East Indian 8 Caucasian	17–32	Neurological examination normal 1 participant on venlafaxine and buproprion	*N* = 11 (all men)	28–56	2 Hispanic 9 Caucasian	57–166	Neurological examination normal 2 participants on amitriptyline
Koldewyn et al. (6) (USA)	Same participants as Hessl et al. (5)									
Adams et al. (7) (USA)	*N* = 38 (8 females, 30 males)	Female: 50.63 ± 11.43 (mean ± SD) Male: 57.20 ± 14.12 (mean ± SD)	NA	Female: 32 ± 6.89 (mean ± SD) Male: 28.42 ± 4.82 (mean ± SD)	No significant difference in psychotropic medication taken	*N* = 88 (16 female with FXTAS, 17 Female without FXTAS, 34 male with FXTAS, 21 male without FXTAS)	Female with FXTAS: 57.50 ± 12.46 (mean ± SD) Female without FXTAS: 44.94 ± 11.23 (mean ± SD) Male with FXTAS: 66.44 ± 6.77 (mean ± SD) Male without FXTAS: 52.38 ± 12.11 (mean ± SD)	NA	Female with FXTAS: 95.19 ± 14.18 (mean ± SD) Female without FXTAS: 96.40 ± 20.17 (mean ± SD) Male with FXTAS: 92.76 ± 18.86 (mean ± SD) Male without FXTAS: 88.52 ± 32.33 (mean ± SD)	75% of females with FXTAS and 41.2% of males with FXTAS on psychotropic medication
Hashimoto et al. (8) (USA)	*N* = 28 (all men)	40–79	NA	17–34	Neurological examination normal	*N* = 55 (31 male with FXTAS, 24 male without FXTAS)	Male with FXTAS: 47–79 Male without FXTAS: 41–78	NA	Male with FXTAS: 59–130 Male without FXTAS: 55–166	NA
Hessl et al. (9) (USA)	*N* = 25 (all men)	30.12 ± 7. 5 (mean ± SD)	68.5% Caucasian	19–33	Neurological examination normal 33.3% Psychoactive medication	*N* = 23 (all men)	32.95 ± 8.89 (mean ± SD)	82.6% Caucasian	55–199	Neurological examination normal 28.6% Psychoactive medication
Selmeczy et al. (10) (USA)	*N* = 48 (all men)	19–76	NA	17–42	Neurological examination normal	*N* = 49 (all men)	18–78	NA	55–199	Neurological examination normal
Brown et al. (11) (UK)	*N* = 17 (all men)	24–68	NA	19–40	Neurological examination normal	*N* = 17 (all men)	24–68	NA	58–185 3 premutation mosaic (150 ± 5; ~200 ± 10), (133, 156; 198 ± 10), (148 ± 5; ~200) 1 Intermediate allele (50 CGGs)	Neurological examination normal

All but one of the seven studies were performed in male population, whereas only one included both male and female permutation carriers. Four of them used functional magnetic resonance imaging (fMRI), five of them used volumetric measures and one used voxel-based morphometry. It is important to note that comorbid medical and psychiatric conditions, such as the mood of participants when performing tasks, were not considered, or at least not mentioned in any of the reviewed studies. Moreover, some of the participants enrolled in the studies were medicated with different drugs (Table 2), which might act as a potential confounding factor, and finally had an effect on the neuroimaging findings.

The synthesis of the 7 neuroimaging studies identified the amygdala and the hippocampus as the two major brain areas involved with FXAND. Table 3 summarizes results reported in the 7 studies.

**Table 3:** Summary of the psychological, neuroimaging and molecular findings reported in *FMR1* premutation carriers compared to control population in the papers reviewed.

**Study**	**Psychological findings**	**Neuroimaging findings**	**Molecular findings**
Hessl et al. (5)	Diminished autonomic responsivity to social–emotional stimuli	Diminished amygdala activation No differences in amygdala volume	Positive correlation with increased *FMR1* mRNA levels
Koldewyn et al. (6)	Significantly worse performance on an immediate memory recall test but not on the in-scanner recall task 24 h later	Reduced left hippocampal activation and increased right parietal activation No differences in hippocampal volume	Positive correlation with increased *FMR1* mRNA levels No significant correlation with the CGG repeat number
Adams et al. (7)	Increased psychological symptoms in *FMR1* premutation carriers with FXTAS (obsessive–compulsive behaviors, somatization and anxiety)	Significant negative correlations between hippocampal volume and psychological symptoms	Negative correlation between CGG repeat size and total and left hippocampal volume in males with FXTAS. Negative correlation between CGG repeat length and right hippocampal volume in females with FXTAS Positive correlation between *FMR1* mRNA levels and left hippocampal volume in female carriers without FXTAS.
Hashimoto et al. (8)	Increased levels of obsessive–compulsiveness and depression Poor working memory performance	Gray matter loss in the left amygdala Decreased gray matter in the left inferior frontal cortex and anterior cingulate cortex	Significant negative effect of CGG repeat size on gray matter density in the dorsomedial frontal regions
Hessl et al. (9)	Higher ratings of communication and reciprocal social behavior symptoms of autism spectrum disorder (althought not significant)	Significantly smaller left and right amygdala volume during an emotion-matching task Reduced right amygdala activation during the task	Reduced FMRP as the primary factor involved in alterations of brain activity and behavior
Selmeczy et al. (10)	No difference on the global severity index score of the SCL-90-R	No significant differences in amygdala volume	Significant negative correlation between amygdala volume and CGG repeat expansions was found in the lower, but not in the higher, range of CGG repeat expansions
Brown et al. (11)	Significantly worse symptoms of neuropsychiatric symptomatology of obsessive-compulsiveness, anxiety, global psychiatric severity and positive symptom distress levels Higher levels of autistic traits Impaired facial emotion recognition	Significantly lower activation at the bilateral superior parietal lobes, bilateral Brodmann Area (BA) 17 (primary visual cortex), right intraparietal area and right BA18 (visual association area) when comparing high and low arousal conditions	Not examined

### Amygdala

The relationship between both the amygdala function and volume with psychological symptoms has been evaluated in several studies.

Regarding the amygdala function, male premutation carriers without FXTAS have been found to have decreased amygdala activation to emotional inputs which correlate with an increase of psychological symptoms. In 2007, Hessl et al. (5) described a negative correlation between psychological symptoms and amygdala activation compared to controls during an fMRI task consistent in passively viewing fearful vs. scrambled faces. Moreover, they also found that *FMR1* premutation participants had decreased potentiation of the eye blink startle reflex to fearful faces, which is an indirect evidence of reduced amygdale activation, and diminished skin conductance response during a brief social stressor; evidencing reduced sympathetic activation. Similarly, Hessl et al. (9) described a negative correlation between higher ratings of autism spectrum symptoms and reduced left amygdala activation during an emotion processing fMRI task.

The reduced amygdala activation in *FMR1* premutation carriers has been associated with abnormal elevation of *FMR1* mRNA (5) and in particular, with reduced FMRP levels (9).

As for the amygdala volume and its correlation with psychological symptoms, results are controversial. While some studies did not find differences between groups in the amygdala volume and psychological symptoms (measured on the SCL-90-R) or cognitive ability (based on full scale IQ) (5, 10), others reported higher ratings of autism spectrum symptoms correlated with smaller bilateral amygdala volume (9). Additionally, Hashimoto et al. (8) demonstrated that increased levels of obsessive–compulsiveness and depression in male premutation carriers was associated with gray matter loss in the left amygdala evaluated by voxel-based morphometry. A plausible explanation for these discordant results might be the different neuroimaging methods used in each study. Whereas, Selmeczy et al. (10) used both 1.5 and 3.0 Tesla MRI, the study conducted by Hessel et al. (9) only used a 3.0 T structural imaging.

Interestingly, even though Selmeczy et al. (10) found no difference between groups in the amygdala volume, a significant negative correlation was found between amygdala volume and the lower range of CGG repeat expansion (CGG≥55 and <85), but not in the higher range of CGG repeat expansion (CGG≥85). This observation raises the intriguing possibility that different molecular mechanisms could be affecting the brain structure, and potentially the function, in *FMR1* premutation carriers depending on the CGG repeat expansion size.

### Hippocampus

The relationship between psychological symptoms and hippocampal function and volume in *FMR1* premutation carriers has also been examined.

Reduced hippocampal activation during an fMRI memory recall task has been associated to psychiatric symptomology in *FMR1* premutation male carriers without FXTAS (6). Moreover, this reduction was found in association with parietal over-activation, which might be a feedback effect to compensate the decreased hippocampal involvement, and abnormal elevation of *FMR1* mRNA.

A hippocampal volume effect has been associated with *FMR1* premutation, albeit some inconsistent findings. While Jäkälä et al. (12) found reduced volumes; Loesch et al. (13) described increased volumes in this region in premutation carriers. On the other hand, Koldewyn et al. (6) did not find hippocampal volume effects associated with the premutation. The lack of consistent findings between these three studies may be due to specific cohort effects, especially if these cohorts included participants with and without FXTAS.

To our knowledge, only two papers explored the relationship between psychological measures and hippocampal size. While Koldewyn et al. (6) did not find hippocampal volume differences, Adams et al. (7) found a significant negative correlation between total hippocampal volume and anxiety in female carriers with and without FXTAS. This association seemed to be mainly driven by the right hippocampus since correlations were stronger. The association in male permutation carriers was weaker and was only significant for one of the psychological problems assessed (paranoid ideation). Furthermore, they found a negative correlation between CGG repeat size and total and left hippocampal volume in males with FXTAS and a similar correlation with CGG repeat length and right hippocampal volume in females with FXTAS (7).

### Other Findings

The selected studies also showed other structural findings in *FMR1* premutation carriers, even though there was no correlation with psychological symptoms. First, total brain volume has been found to be significantly decreased in older permutation carriers (13). Secondly, gray matter loss has also been examined in several studies: all of them evidencing reduced density in several brain regions in the permutation group. In this regard, Hashimoto et al. (8) reported significant gray matter loss in medial temporal lobe structures and cerebellar areas, such as the vermis lobule VII, and the cerebellar hemisphere lobule IX, as well as in multiple regions outside the cerebellum that have been related with several psychiatric conditions. Although significant loss was found when comparing the permutation group with controls, the correlation analysis failed to show significant association between all these areas and psychiatric problems in *FMR1* premutation carriers. Finally, Brown et al. (11) found that permutation carriers exhibited significantly lower BOLD activation compared to controls at the bilateral superior parietal lobe, bilateral Brodmann Area (BA) 17 (V1) (primary visual cortex), right intraparietal area, and right BA18 (V2) (visual association area), when comparing high and low arousal conditions. However, no correlations were found between more psychiatric symptoms and higher levels of autistic traits observed in carriers and BOLD activation at the emotional processing fMRI task.

### Study Limitation

Overall, the studies herein reviewed provided valuable neuroimaging data of brain abnormalities in *FMR1* premutation carriers related to neuropsychiatric disorders. However, the majority of them were conducted on small sample sizes of groups, which might have limited detection of true significances. Moreover, some of them included *FMR1* premutation carriers with FXTAS which might also influence significant results. In addition, the neuroimaging methods used were different, which makes it difficult to compare results. It should also be noted that fMRI is a complex technique that can be influenced by many factors such as the paradigm design (the manner of stimulating the brain in order to obtain meaningful information), magnetic field strength, MRI acquisition parameter and subject collaboration. Furthermore, the parameters that have an influence in blood flow and oxygenation have an impact on fMRI signal and, overall, fMRI has a challenging data interpretation (14). Finally, there are several variables that have to be taken into account as potential confounding factors in all the studies reviewed. Aspects such as comorbid medical conditions, medication taken by the participants (psychotropic or psychoactive) or unmeasured (unobserved) factors, such as the mood or the stress of participants, could have influenced the results. The reviewed studies were aware of these aspects and tried to minimize their effect by matching *FMR1* premutation and control groups, although this was not always possible.

## Discussion

*FMR1* premutation carriers are at risk of developing an adult-onset neurodegenerative disorder named FXTAS (20). In addition, several studies reveal that young *FMR1* premutation carriers are at increased risk for psychiatric conditions, memory problems and executive deficits (15–18). Indeed, brain function is also affected by *FMR1* premutation status in relatively young premutation carriers without FXTAS who demonstrate no overt neurological symptoms (5, 6, 9, 11). Contrary to movement-related neurodegeneration, which increases over time, emotional symptoms seem to be consistent over the lifespan in *FMR1* premutation carriers; suggesting a neurodevelopmental origin, different from the neurodegeneration seen in FXTAS (7, 11, 19).

*FMR1* premutation carriers have elevated levels of *FMR1* mRNA and can also have some degree of FMR1 protein (FMRP) deficiency, mainly at the high end of the *FMR1* premutation range (20). FMRP is an RNA binding protein that regulates the translation of many gene products and has been implicated in dendritic maturation and in the formation of axons and myelin (21–23). In fact, FMRP activity is regulated in response to neuronal activity, and is an important mediator of synapse development, synaptic plasticity, learning and memory (reviewed in (52)). The mRNA targets of FMRP have received additional attention due to their enrichment for genes harboring risk to psychiatric disorders (24). Recently, Clifton et al. (52) has reported that a substantial proportion of FMRP targets have functions related to synaptic activity, anatomy or development. The association between synaptic plasticity and psychiatric disorders has been well-established with several genetic and functional studies describing the relevance of imbalanced of excitation and inhibition (25). FMRP levels have been reported to be reduced in *FMR1* premutation brains of a mouse model (26), as well as in patients with psychiatric disorders such as autism, schizophrenia, bipolar disorder, and major depressive disorder (27). Whilst some studies had found a relationship between *FMR1* mRNA levels and psychiatric symptomology or brain function in *FMR1* premutation carriers (5, 6, 28), others have pointed out a stronger relationship with reduced FMRP levels (9). Taking into consideration the importance of FMRP in normal neurodevelopment and its association with psychiatric disorders, there is the possibility that moderate reductions in FMRP levels could play a role in the behavioral dysfunction seen in *FMR1* premutation carriers.

Both structural and functional changes in the hippocampus and amygdala have been found to be altered in *FMR1* premutation carriers and some studies have proved a relationship between such changes and psychiatric symptomatology (5–9). However, while functional changes have been consistently reported (5, 6, 9, 11), volumetric measures showed some inconsistent results, with some studies showing increase, decrease, or no significant differences between hippocampal and amygdala volumes or voxel density in *FMR1* premutation carriers (6, 10, 12, 13, 29–31). The lack of consistent findings between studies may be due to a cohort effect either in the size, the inclusion of participants with and without FXTAS, gender of participants or differences in the CGG repeat size. Moreover, technical aspects such as the volumetric techniques used, the image quality or segmentation technique followed might also contribute to explain discrepancies. However, and in consonance, findings of amygdala and hippocampal volumes in mood disorders such as depression or anxiety in non *FMR1* premutation carriers have also been conflicting, with some studies reporting positive, negative and no associations (32–46).

Although an association between the above described structural and functional changes and molecular aspects of the *FMR1* pemutation carriers has been proven in some of the studies reported (Table 3), there is still need to better define them. What does seem certain, and evidence points to it, is that the limbic system is a brain structure particularly susceptible to RNA toxicity. During normal fetal development, the hippocampus is one of the areas in which *FMR1* transcription is the highest (47) and in adult human brain, the hippocampus demonstrates one of the highest expression rates of *FMR1* mRNA (48). Similarly, *FMR1* mRNA levels are disproportionately increased in the amygdala of *FMR1* premutation carriers (48, 49). Moreover, in post-mortem brain studies of male *FMR1* premutation carriers with FXTAS, it has been shown that, compared to other brain areas, the hippocampi harbors high density of intranuclear inclusions (22, 23). Additionally, the knock-in mouse model of the *FMR1* premutation showed a significantly reduced FMRP expression in several brain regions, including the hippocampus (50).

Future studies with larger and more homogeneous sample size are needed in order to increase statistical power and validate such findings. Furthermore, longitudinal studies will be needed to evaluate progression of the neuroimaging and clinical findings. In addition, looking for modifying factors, either predisposing or protective factors, able to modulate neuroimaging, and clinical symptomatology is a key point for the knowledge and understanding of the disease. It is also crucial to clarify the metabolic causes of brain toxicity and to identify early presymptomatic brain changes that precede, but which are ultimately associated with neuropsychiatry disorders. Finally, further investigations that include quantitative measurements of molecular changes will be of great interest in order to clarify the relative roles of increased *FMR1* mRNA and FMRP protein changes in the Fragile X-associated phenotypes.

Overall this review would like to encourage all *FMR1* research groups furthering investigating the neuropsychiatric involvement in *FMR1* premutation with testing other brain systems, with additional probes of brain function and a broader scope of psychiatric symptom measurement. The combination of all these missing data would help to obtain a comprehensive landscape of the neuropsychiatric phenotype associated with the *FMR1* premutation

## Data Availability Statement

The original contributions presented in the study are included in the article/Supplementary Files, further inquiries can be directed to the corresponding author/s.

## Author Contributions

All authors listed have made a substantial, direct and intellectual contribution to the work, and approved it for publication.

## Funding

This work was supported by the Instituto de Salud Carlos III (PI17/01067), co-financed by Fondo Europeo de Desarrollo Regional (FEDER) una manera de hacer Europa AGAUR from the Autonomous Catalan Government (2017SGR1134). The CIBER de Enfermedades Raras is an initiative of the Instituto de Salud Carlos III.

## Conflict of Interest

The authors declare that the research was conducted in the absence of any commercial or financial relationships that could be construed as a potential conflict of interest.

## Publisher's Note

All claims expressed in this article are solely those of the authors and do not necessarily represent those of their affiliated organizations, or those of the publisher, the editors and the reviewers. Any product that may be evaluated in this article, or claim that may be made by its manufacturer, is not guaranteed or endorsed by the publisher.
