# Supplementary material for: Neuroimaging Insight Into Fragile X-Associated Neuropsychiatric Disorders: Literature Review
Source: Front Psychiatry. 2021 Oct 15;12:728952. doi: 10.3389/fpsyt.2021.728952 (PMC8554234; doi:10.3389/fpsyt.2021.728952)
Supplement: Supplementary file 1 [file Table_1.DOCX]

**Supplementary Materials**

Supplementary Table 1. Search terms used in the study.

| FXAND  FXANC  FragileX-associated neuropsychiatric disorders  Psychiatric symptoms | FMR1 premutation  Fragile X premutation | MRI  Imaging  Neuroimaging  Functional studies  fMRI  Tomography  Diffusion tensor imaging  DTI  Spectroscopy |
| --- | --- | --- |
| Anxiety  Depression/depressive  Obsessive compulsive disorder  OCD  ADHD/ Attention Deficit Disorder with Hyperactivity  Substance abuse/ Substance-Related Disorders |  | Amygdala  Insula  Thalamus  Hippocampus  Cingulate cortex  [Caudate](https://radiopaedia.org/articles/caudate-nucleus?lang=us)  CSTC  Basal ganglia  Putamen  Globus pallidus  Caudate  White matter  Grey matter  Gray matter  Cortical thickness  Gyrification  Gyrus  Cerebellum  Precuneus  Striatum  Accumbens |

**Search strategy**

PubMed, Web of Science, PsycINFO and Cochrane Central Register of Controlled Trials were searched for eligible studies from 2000 up until April 2021.

**PUBMED:**

(FXAND OR FXANC OR Fragile X-associated neuropsychiatric disorders OR Psychiatric symptom* OR anxiety OR depression OR depressive OR Obsessive compulsive disorder OR OCD OR ADHD OR Attention Deficit Disorder with Hyperactivity OR Substance abuse OR Substance-Related Disorders) AND (FMR1 premutation OR Fragile X premutation OR FMR1 disorders) AND (MRI OR magnetic resonance imaging OR Neuroimaging OR Functional studies OR Tomography OR Diffusion tensor imaging OR DTI OR Spectroscopy OR Amygdala OR Insula OR Thalamus OR Hippocampus OR Cingulate cortex OR [Caudate](https://radiopaedia.org/articles/caudate-nucleus?lang=us) OR CSTC OR Basal ganglia OR Putamen OR Globus pallidus OR Caudate OR White matter OR Grey matter OR Gray matter OR Cortical thickness OR Gyrification* OR Gyrus OR Cerebellum OR Precuneus OR Striatum OR Accumbens ). PUBMED results: 298

(Fragile X OR Fragile X-associated neuropsychiatric disorders OR Psychiatric symptom* OR anxiety OR depression OR depressive OR Obsessive compulsive disorder OR OCD OR ADHD OR Attention Deficit Disorder with Hyperactivity OR Substance abuse OR Substance-Related Disorders ) AND (FMR1 premutation OR Fragile X premutation OR FMR1 disorders ) AND (MRI OR magnetic resonance imaging OR Neuroimaging OR Functional studies OR Tomography OR Diffusion tensor imaging OR DTI OR Spectroscopy OR Amygdala OR Insula OR Thalamus OR Hippocampus OR Cingulate cortex OR [Caudate](https://radiopaedia.org/articles/caudate-nucleus?lang=us) OR CSTC OR Basal ganglia OR Putamen OR Globus pallidus OR Caudate OR White matter OR Grey matter OR Gray matter OR Cortical thickness OR Gyrification OR Gyrus OR Cerebellum OR Precuneus OR Striatum OR Accumbens ). PUBMED: results: 1480

fragile x associated neuropsychiatric conditions fmr1 premutation

"fragile"[All Fields] AND "x"[All Fields] AND ("associate"[All Fields] OR "associated"[All Fields] OR "associates"[All Fields] OR "associating"[All Fields] OR "association"[MeSH Terms] OR "association"[All Fields] OR "associations"[All Fields]) AND ("neuropsychiatric"[All Fields] OR "neuropsychiatrically"[All Fields] OR "neuropsychiatrics"[All Fields]) AND ("condition s"[All Fields] OR "conditions"[All Fields] OR "disease"[MeSH Terms] OR "disease"[All Fields] OR "condition"[All Fields]) AND "fmr1"[All Fields] AND ("premutated"[All Fields] OR "premutation"[All Fields] OR "premutations"[All Fields])Translations associated: "associate"[All Fields] OR "associated"[All Fields] OR "associates"[All Fields] OR "associating"[All Fields] OR "association"[MeSH Terms] OR "association"[All Fields] OR "associations"[All Fields] neuropsychiatric: "neuropsychiatric"[All Fields] OR "neuropsychiatrically"[All Fields] OR "neuropsychiatrics"[All Fields]

conditions: "condition's"[All Fields] OR "conditions"[All Fields] OR "disease"[MeSH Terms] OR "disease"[All Fields] OR "condition"[All Fields]

premutation: "premutated"[All Fields] OR "premutation"[All Fields] OR "premutations"[All Fields]. PUBMED: results 1171

**ISI WEB**

TEMA: [(‎Fragile X-associated neuropsychiatric disorders OR Fragile X-associated Neuropsychiatric Conditions OR Psychiatric symptom*) OR anxiety) OR depression) OR depressive) OR Obsessive compulsive disorder) OR OCD) OR ADHD) OR Attention Deficit Disorder with Hyperactivity) OR Substance abuse) OR Substance-Related Disorders)AND TEMA: ‎(‎FMR1 permutation OR Fragile X permutation) OR FMR1 disorders) AND TEMA: ‎(‎MRI OR magnetic resonance imaging) OR Neuroimaging) OR Functional studies) OR Tomography) OR Diffusion tensor imaging) OR DTI) OR Spectroscopy) OR Amygdala) OR Insula) OR Thalamus) OR Hippocampus) OR Cingulate cortex) OR Caudate) OR CSTC) OR Basal ganglia) OR Putamen) OR Globus pallidus) OR Caudate) OR White matter) OR Grey matter) OR Gray matter) OR Cortical thickness) OR Gyrification*) OR Gyrus) OR Cerebellum) OR Precuneus) OR Striatum) OR Accumbens)](https://apps-webofknowledge-com.sire.ub.edu/UseSpellSuggestion.do?action=takeSuggestion&product=UA&SID=D3GuaKna7sNRzpICdlh&search_mode=GeneralSearch&update_back2search_link_param=yes&viewType=summary&qid=4)

[TEMA: (‎FXAND OR FXANC) OR Fragile X-associated neuropsychiatric disorders) OR Psychiatric symptom*) OR anxiety) OR depression) OR depressive) OR Obsessive compulsive disorder) OR OCD) OR ADHD) OR Attention Deficit Disorder with Hyperactivity) OR Substance abuse) OR Substance-Related Disorders) AND TEMA: ‎(‎FMR1 permutation OR Fragile X permutation) OR FMR1 disorders) AND TEMA: (‎MRI OR magnetic resonance imaging) OR Neuroimaging) OR Functional studies) OR Tomography) OR Diffusion tensor imaging) OR DTI) OR Spectroscopy) OR Amygdala) OR Insula) OR Thalamus) OR Hippocampus) OR Cingulate cortex) OR Caudate) OR CSTC) OR Basal ganglia) OR Putamen) OR Globus pallidus) OR Caudate) OR White matter) OR Grey matter) OR Gray matter) OR Cortical thickness) OR Gyrification*) OR Gyrus) OR Cerebellum) OR Precuneus) OR Striatum) OR Accumbens))](https://apps-webofknowledge-com.sire.ub.edu/UseSpellSuggestion.do?action=takeSuggestion&product=UA&SID=D3GuaKna7sNRzpICdlh&search_mode=GeneralSearch&update_back2search_link_param=yes&viewType=summary&qid=6)

ISI WEB: results 244 (+ FXAND OR FXANC same results) and: articles, other, review, case report.

**PSCHYNFO**

(FXAND OR FXANC OR Fragile X-associated neuropsychiatric disorders OR Psychiatric symptom* OR anxiety OR depression OR depressive OR Obsessive compulsive disorder OR OCD OR ADHD OR Attention Deficit Disorder with Hyperactivity OR Substance abuse OR Substance-Related Disorders ) AND( FMR1 premutation OR Fragile X premutation OR FMR1 disorders ) AND ( MRI OR magnetic resonance imaging OR Neuroimaging OR Functional studies OR Tomography OR Diffusion tensor imaging OR DTI OR Spectroscopy OR Amygdala OR Insula OR Thalamus OR Hippocampus OR Cingulate cortex OR Caudate OR CSTC OR Basal ganglia OR Putamen OR Globus pallidus OR Caudate OR White matter OR Grey matter OR Gray matter OR Cortical thickness OR Gyrification* OR Gyrus OR Cerebellum OR Precuneus OR Striatum OR Accumbens ). PSCHYNFO: results 23

**COCHRANE**

fmr1 premutation in Title Abstract Keyword OR Fragile X premutation*

Cochrane: results: 5 trials. Title Abstract Keyword - (Word variations have been searched). Limit: results 5

"fragile X syndrome" in Title Abstract Keyword AND imaging in Title Abstract Keyword OR neuroimaging in Title Abstract Keyword - (Word variations have been searched). Limit: results 6

(FXAND OR FXANC OR Fragile X-associated neuropsychiatric disorders OR Psychiatric symptom* OR anxiety OR depression OR depressive OR Obsessive compulsive disorder OR OCD OR ADHD OR Attention Deficit Disorder with Hyperactivity OR Substance abuse OR Substance-Related Disorders ) AND ( FMR1 premutation OR Fragile X premutation OR FMR1 disorders ) AND ( MRI OR magnetic resonance imaging OR Neuroimaging OR Functional studies OR Tomography OR Diffusion tensor imaging OR DTI OR Spectroscopy OR Amygdala OR Insula OR Thalamus OR Hippocampus OR Cingulate cortex OR Caudate OR CSTC OR Basal ganglia OR Putamen OR Globus pallidus OR Caudate OR White matter OR Grey matter OR Gray matter OR Cortical thickness OR Gyrification* OR Gyrus OR Cerebellum OR Precuneus OR Striatum OR Accumbens )" Limits: results 2
